# Supplementary material for: Interactions of EGFR/PTEN/mTOR-Pathway Activation and Estrogen Receptor Expression in Cervical Cancer
Source: J Pers Med. 2023 Jul 26;13(8):1186. doi: 10.3390/jpm13081186 (PMC10455725; doi:10.3390/jpm13081186)
Supplement: Supplementary file 1 [file jpm-13-01186-s001.zip › jpm-2514109-supplementary.pdf]

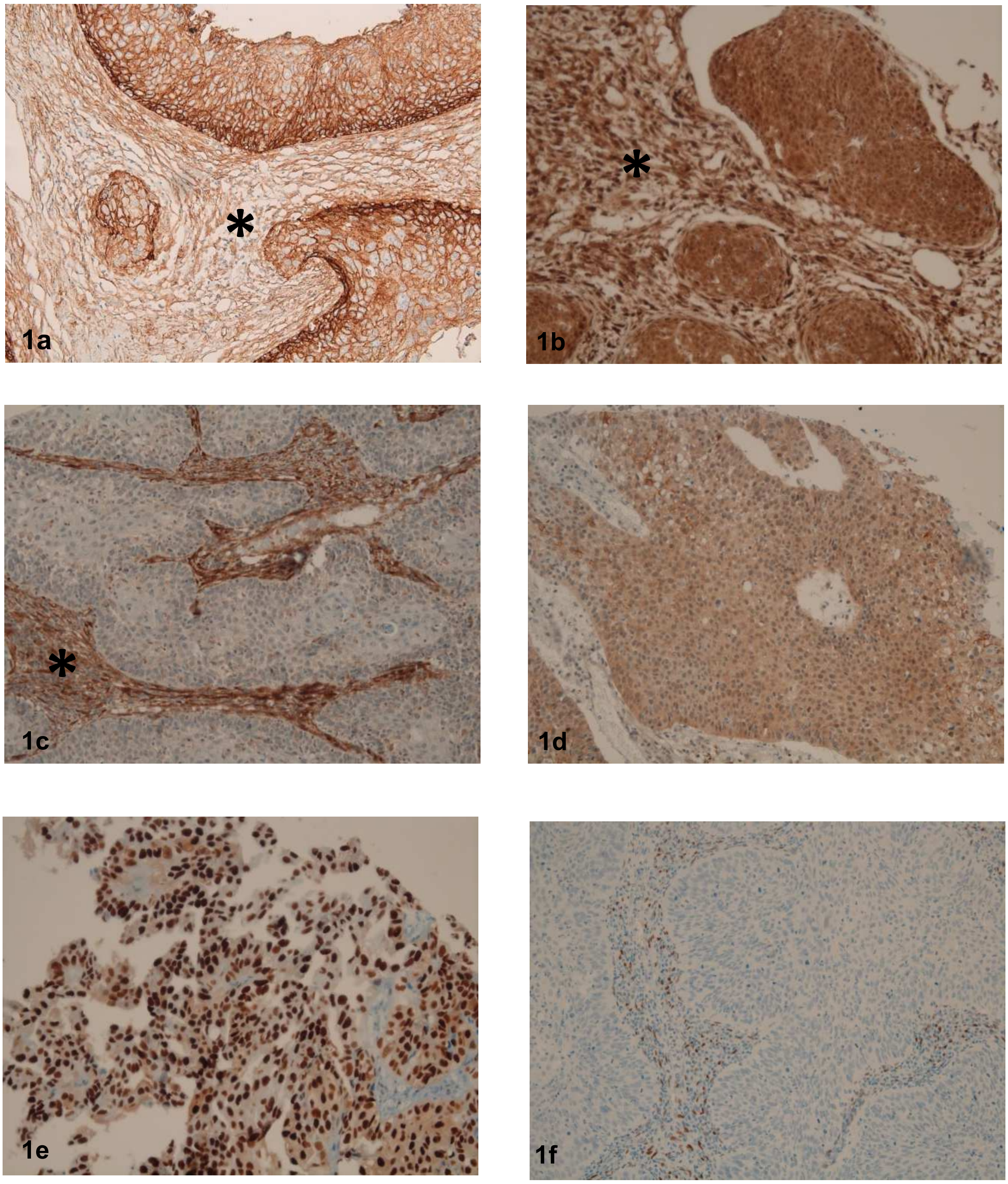

Supplement Figure S1a- 1f. Expression of EGFR (1a), PTEN (1b and c), mTOR (d), ER (e), PR (f) in cancer cells and tumor surrounding cells (*). Figure 1b shows expression of PTEN in cervical cancer and in tumor surrounding stromal cells, figure 1c shows a case of PTEN expression in in tumor surrounding stromal cells (*), but no expression in cervical cancer cells. In figure 1f, no progesterone expression could be observed. (original magnification 20x)
